# The sequence flanking the N-terminus of the CLV3 peptide is critical for its cleavage and activity in stem cell regulation in *Arabidopsis*

**DOI:** 10.1186/1471-2229-13-225

**Published:** 2013-12-27

**Authors:** Ting-Ting Xu, Xiu-Fen Song, Shi-Chao Ren, Chun-Ming Liu

**Affiliations:** 1 Key Laboratory of Plant Molecular Physiology, Institute of Botany, Chinese Academy of Sciences, Nanxincun 20, Fragrant Hill, Beijing 100093, China; 2 University of Chinese Academy of Sciences, Beijing 100049, China

**Keywords:** CLV3, Peptide cleavage, Flanking sequence, AA substitution, Stem cell regulation

## Abstract

**Background:**

Although it is known that CLAVATA3 (CLV3) acts as 12- and/or 13-amino acid (AA) secreted peptides to regulate the number of stem cells in shoot apical meristems (SAMs), how functional CLV3 peptides are generated and if any particular sequences are required for the processing remain largely unknown.

**Results:**

We developed a mass spectrometry (MS)-based *in vitro* assay to monitor the cleavage of heterologously produced CLV3 fusion protein. Through co-cultivation of the fusion protein with *Arabidopsis* seedlings, we identified two cleavage sites: the previously reported one before Arg70 and a new one before Met39. Using synthetic peptides together with MALDI-Tof-MS analyses, we demonstrated that the non-conserved 5-AA motifs flanking N-termini of the CLV3 and its orthologous CLE1 peptides were critical for their cleavages and optimal activities *in vitro*. We also found that substitutions of Leu69 by Ala in fusion protein and in synthetic peptide of CLV3 compromised their cleavages, leading to significantly reduced activities in regulating the sizes of shoot and root meristems.

**Conclusions:**

These results suggest that 5-AA residues flanking the N-terminus of CLV3 peptide are required for proper cleavages and optimal function in stem cell regulation.

## Background

It has been known for a long time that small peptides act as endocrinal hormones and neurotransmitters to facilitate intercellular communications in animals [1-4]. In plants since the first peptide hormone, systemin, is discovered twenty years ago [5], many small peptides have been identified, regulating developmental and defense processes including pollen tube guidance [6,7], microspore-tapetum interaction [8,9], stomata patterning [10-13], cell proliferation [14,15], stem cell homeostasis [16-18] and wounding responses [5,19,20].

CLV3 is one of the well-studied peptide hormones in plants. The *CLV3* gene encodes a 96-AA secretory protein that functions in a feedback regulation loop to restrict the number of stem cells in SAMs by repressing the expression of the *WUSCHEL* (*WUS*) transcription factor [16,21]. Combined genetic and biochemical analyses show that CLV3 acts through interacting with leucine-rich repeat (LRR) receptor kinases of CLAVATA1 (CLV1) and Receptor Protein Kinase 2 (RPK2), a CLAVATA2 (CLV2) receptor-like protein, and a membrane-bound Suppressor Of LLP1 2 (SOL2)/CORYNE (CRN) protein [22-25]. CLV3 shares a conserved 14-AA CLE motif with a large number of CLE proteins [26,27]. Domain deletion analyses reveal that most of the non-conserved sequence located between the secretion signal peptide and the CLE motif, and the sequence after the CLE motif are not required for the CLV3 function *in vivo*[28]. *In vitro* experiments show that synthetic 12-, 13- and 14-AA peptides, namely CLV3p12, CLV3p13 and CLV3p14, corresponding to the CLE motif are functional in promoting stem cell differentiation in both shoot and root meristems (RMs) [17,28-31]. Direct interaction between CLV3p12 and CLV1 has been demonstrated in tobacco BY-2 cells [32]. MALDI-Tof MS analyses of transgenic calli over-expressing *CLV3* identified a 12-AA hydroxylated peptide [29], while more recently nano-LC-MS analyses of *Arabidopsis* seedlings over-expressing *CLV3* identified a 13-AA arabinose-glycosylated peptide [30]. These two candidate endogenous CLV3 peptides share the same N-terminal Arg70 (the number refers to the residue in the full-length CLV3 protein) and hydroxylation modifications on Pro73 and Pro76. Although Ala-substitution experiments showed that the glycosylation on Pro76 is not required for CLV3 functions in SAMs [33], analyses of chemically synthesized CLV3 glycopeptides indicate that the glycosylation may contribute to the stability of the peptide [34]. Using a heterologously produced GST-tagged CLV3 fusion protein in combination with extracts of cauliflower and tobacco BY-2 cells, two cleavage sites, one before Met42 and one before Arg70, are detected by gel blot and MS analyses [35,36]. For nematode GrCLE1, cleavages before Arg130, Arg151, Arg172 and Arg193 have been observed using cauliflower and potato root extracts [37]. In *Medicago truncatula,* it has been shown that MtCLE36 is processed by enzymes in extracellular fluids, producing a 15-AA peptide, SKRRVPNGPDPIHNR [38]. Although these studies provide basic knowledge on the cleavage of CLE peptides, sequences involved in the cleavage recognition and how such cleavages contribute to the activity of these peptides remain elusive.

In animals, enzymes involved in peptide cleavages are serine endopeptidase sub-family subtilases (SBTs), also known as subtilisin-like proprotein convertases [39]. The cleavage generally occurs between or after dibasic Lys/Arg-Arg residues, or after the monobasic residue Arg, releasing peptides with N- and/or C-terminal basic residues [40]. These basic residues are then removed by exopeptidases such as carboxypeptidase E (CPE) [41]. In plants most SBTs are predicted to function in extracellular spaces, based on the presence of an N-terminal secretion signal sequence [42]. In *Arabidopsis*, AtSBT1.1 and AtSBT6.1 have been shown to cleave PSK4 and RALF23, respectively [43,44], suggesting a conserved peptide processing mechanism between animals and plants.

A conventional way to study peptide cleavage is through gel-based assays in combination with heterologously produced peptidases [45,46]. Due to the low sensitivity of the method, it is often difficult to detect small peptide fragments. The peptidomic technology developed in recent years has the potential to solve this problem [47-50]. As an example, peptidomic comparison between a mouse mutant defective in carboxypeptidase E (CPE) and the wild-type revealed a role of the carboxypeptidase in neuropeptide processing [51]. In *Medicago truncatula,* peptidomic technology has been applied to identify small peptides, although most peptides detected seem to be degradation products from abundant proteins such as ribosomal proteins and histones [52].

By taking advantage of the well-studied CLV3, we established a MS-based peptidomic method to examine peptide fragments cleaved by enzymes secreted from intact *Arabidopsis* seedlings. Using the method we identified two internal cleavage sites in the CLV3 proprotein, one before Met39 and another one before Arg70. Further, we demonstrated that the Leu69 and 5-AA residues flanking the N-terminus of the CLV3 peptide are important for the cleavage before Arg70 and for the optimal activity *in vitro*.

## Results

### Heterologously produced CLV3 fusion protein is active *in vitro*

**Figure 1:**
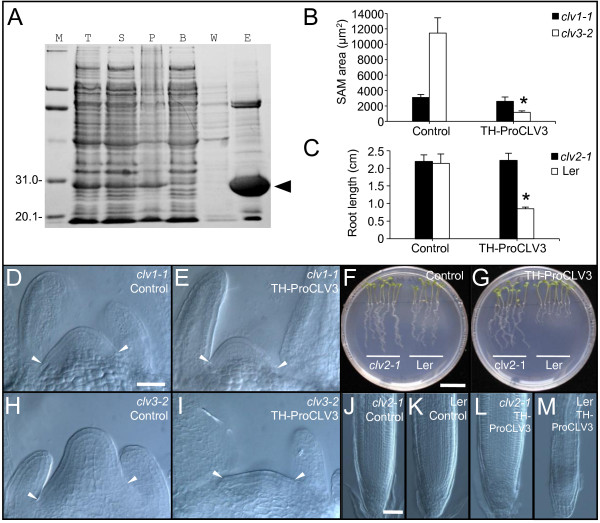
**Purification and *in vitro *assays of the TH-ProCLV3 fusion protein. (A)** Affinity purification of TH-ProCLV3. M, molecular weight marker; T, total cell lysate; S, supernatant; P, pellet; B, efflux with binding buffer; W, efflux with washing buffer; E, efflux with elution buffer; arrowhead, TH-ProCLV3. **(B)** Sizes of SAMs of 8-d-old *clv1-1* and *clv3-2* seedlings (n = 8) after the treatment with or without 1 μM TH-ProCLV3. Error bar = ± SD. The asterisk indicates significant differences (*P* < 0.01 by Student’s *t*-test) between SAM sizes of *clv3-2* seedlings with and without TH-ProCLV3 treatments. **(C)** Root lengths of 8-d-old L*er* and *clv2-1* seedlings treated with or without 1 μM TH-ProCLV3 (n = 8). Error bar = ± SD. The asterisk indicates significant differences (*P* < 0.01 by Student’s *t*-test) between root lengths of L*er* seedlings with and without TH-ProCLV3 treatment. **(D, E)** SAMs of *clv1-1* seedlings treated with **(E)** or without **(D)** 1 μM TH-ProCLV3. **(H, I)** SAMs of *clv3-2* seedlings treated with **(I)** or without **(H)** 1 μM TH-ProCLV3. Note the reduced sizes of the SAMs in *clv3-2* seedlings after the treatment with TH-ProCLV3. Arrowheads point to the margins of the SAMs. **(F, G)** Eight-d-old *clv2-1* and L*er* seedlings treated with **(G)** or without **(F)** 1 μM TH-ProCLV3. **(J-M)** Root tips of *clv2-1***(J, L)** and L*er***(K, M)** seedlings after incubation with **(J, K)** or without **(L, M)** 1 μM TH-ProCLV3. Bar in **D** = 50 μm for **D**, **E**, **H** and **I**; Bar in **F** = 1 cm for **F** and **G**; Bar in **J** = 50 μm for **J** to **M**.

For the production of the CLV3 proprotein (without the secretion signal peptide), a construct was made in which tandem aligned Trx and His tags were fused to the N-terminus of CLV3 (named *TH-ProCLV3*). The construct was transformed to *E. coli* strain BL21(DE3) to produce the TH-ProCLV3 fusion protein with a total molecular mass of 23.8 kD. The TH-ProCLV3 was then purified by affinity chromatography using a Ni-NTA agarose column (Figure 1A), and quantified by Bradford assay [53] after dialyzed using a 7-kD cut-off dialysis bag.

*In vitro* activity assays of the TH-ProCLV3 fusion protein in SAMs were performed using *clv3-2* and *clv1-1* (both in L*er* background) mutants of *Arabidopsis*, as described previously [28]. We observed that the incubation of *clv3-2* seedlings with 1 μM TH-ProCLV3 for 8 days led to significant reduced sizes of SAMs (Figure 1B, H, and I), while no evident reduction was observed in SAMs of *clv1-1* seedlings (Figure 1B, D, and E), suggesting that the TH-ProCLV3 fusion protein produced in *E. coli* is active in restricting SAMs in a CLV1-dependent manner.

Similarly, *in vitro* root assays [17] were performed in L*er* and *clv2-1* (in L*er* background) seedlings. We observed that root growths in L*er* seedlings treated with TH-ProCLV3 were inhibited greatly (Figure 1C, F, and G), as previously reported in the CLV3 peptide treatment [31], while L*er* seedlings grown on media without the fusion protein showed normal root growth (Figure 1C, F, and G). In contrast, no evident growth inhibition was observed in *clv2-1* seedlings grown on media with TH-ProCLV3 (Figure 1C, F, and G). Microscopic examinations revealed that the TH-ProCLV3 treatment resulted in termination of RMs in L*er*, but not in *clv2-1* (Figure 1J-M). These results together suggest that the TH-ProCLV3 fusion protein produced in *E. coli* is active in regulating the sizes of SAMs and RMs.

### Detection of cleavages in CLV3 fusion protein

To analyze the cleavage of CLV3, we developed a MALDI-Tof MS-based method to profile peptide fragments produced *in vitro*. Pre-germinated L*er* seedlings were inoculated with the TH-ProCLV3 fusion protein in a liquid medium, then the media were harvested at different time points and subjected directly to MALDI-Tof MS analyses. The fusion protein inoculated in the same medium without seedlings was used as a control. We observed that the optimal time for analyzing peptide cleavage was 24 hr after the incubation with L*er* seedlings, when abundant small peptides with different molecular masses were detected (Additional file 1).

**Figure 2:**
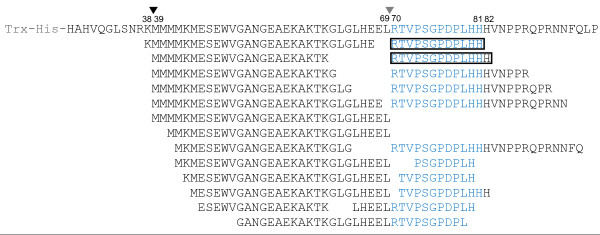
**Peptide fragments produced from ProCLV3 after incubations.** Matching fragments detected are shown below the ProCLV3 sequence, as identified with Q-Tof MS/MS analyses. The core CLE motif (corresponding to the CLV3 peptide) of CLV3 is shown in blue. The CLV3p12 and CLV3p13 peptides are framed. Two internal cleavage sites, before Met39 and before Arg70, are indicated by arrowheads.

To obtain exact sequences of these fragments, Q-Tof MS/MS analyses were performed in these samples. SEQUEST searches for peptide sequences matching the CLV3 proprotein allowed us to identify over 20 peptides (Figure 2). Among them, both the CLV3p12 and the CLV3p13 peptides, the backbones of two candidate forms of mature CLV3 peptides [29,30], were included (Figure 2). After alignment of these fragments with the CLV3 proprotein, two most frequently occurring termini, N-terminal Met39 and Arg70, were detected (Figure 2), suggesting internal cleavage sites located before Met39 and before Arg70. The cleavage before Arg70 has been previously reported [36], however, the one before Met39 is novel. Since Met39 is located in the non-functional region [28], the cleavage might be not functional relevant.

It should be noted that, although peptides with C-terminal His81 and His82 were detected, no peptides with matching N-terminal His82 and Val83 were found. Instead, we detected peptides with variable C-termini. It is plausible that no specific internal cleavage site(s) are present in this region, while carboxypeptidases are involved in processing the C-terminus of the CLV3. Recently, Tamaki *et al.* showed that an endosome-localized SOL1 carboxypeptidase is responsible for removing the C-terminal Arg residue from the CLE19 proprotein [54]. Since intact L*er* seedlings were used in our assay, the C-terminal processing activities detected may come from non-specific extracellular carboxypeptidases instead of SOL1.

### N-terminal flanking sequence affected the cleavage of CLV3

**Figure 3:**
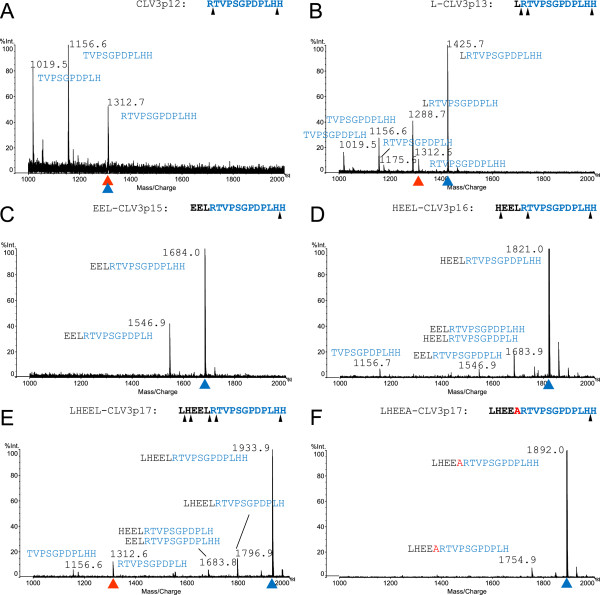
**MALDI-Tof MS analyses of CLV3 peptides with N-terminal extensions.** Spectra of CLV3p12 **(A)**, L-CLV3p13 **(B)**, EEL-CLV3p15 **(C)**, HEEL-CLV3p16 **(D)**, LHEEL-CLV3p17 **(E)**, and LHEEA-CLV3p17 **(F)** after 3-d incubations with L*er* seedlings. Sequences of the original peptides are shown on the upper right of each figure, and the observed cleavage sites are marked by black arrowheads; sequences of fragments are labeled next to corresponding peaks. Original peptides, blue arrowheads; CLV3p12, red arrowheads. The Ala substitution in **F** is in red.

To examine if residues flanking the N-terminus of the CLV3 peptide are required for internal cleavage, we chemically synthesized the CLV3p12 and peptides with additional 1, 3, 4 or 5 residues, namely L-CLV3p13, EEL-CLV3p15, HEEL-CLV3p16 and LHEEL-CLV3p17. These peptides were inoculated in liquid media with *Arabidopsis* (L*er*) seedlings, and medium samples were harvested at different time points and subjected directly to MALDI-Tof MS analyses. We observed that the optimal time for analyzing the cleavage of these peptides was 3 d after the inoculation, when abundant cleaved fragments were detected (Additional file 2). For CLV3p12 and L-CLV3p13 we observed a gradual removal of residues from both N- and C-termini (Figure 3A and B). For EEL-CLV3p15, removal of the C-terminal His residue was observed, but not N-terminal residues (Figure 3C). No internal cleavages for CLV3p12, L-CLV3p13 and EEL-CLV3p15 were detected. In contrast, in addition to gradual removal of terminal residues for HEEL-CLV3p16 and LHEEL-CLV3p17, internal cleavages were also observed (Figure 3D and E). In particular, for HEEL-CLV3p16, a cleavage before Thr71 was observed, producing an 11-AA peptide that is expected to be non-functional [28], while for LHEEL-CLV3p17, cleavages before Arg70 and Thr71 were detected, producing the functional CLV3p12 and a non-functional 11-AA peptide, respectively (Figure 3E). As such, it is possible that the internal cleavage before Arg70 requires at least 5 flanking residues, while exopeptidases are involved in removing residues from both termini.

### CLV3 peptide with five additional N-terminal flanking residues showed similar activity as CLV3p12

**Figure 4:**
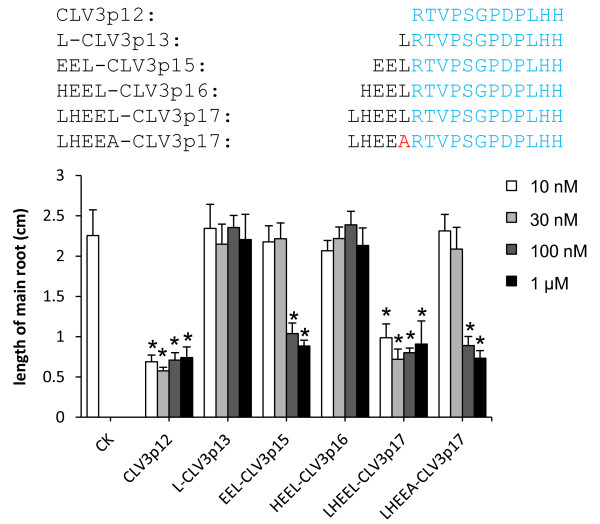
***In vitro *root assays of CLV3 peptides with N-terminal extensions.** Average root lengths of 8-d-old L*er* seedlings (n = 8) after incubations on media containing CLV3 peptides with different N-terminal extensions. Error bar = ± SD. Aligned sequences of all peptides are shown. The Ala substitution in the LHEEA-CLV3p17 is highlighted in red. Average root lengths with significant differences from the non-treated one (*P* < 0.01 by Student’s *t*-test) are marked with asterisks.

To evaluate activities of above-mentioned synthetic peptides with various N-terminal extensions, we performed *in vitro* root assays using L*er* seedlings. The lengths of primary roots were measured after 8-d inoculations with either CLV3p12, L-CLV3p13, EEL-CLV3p15, HEEL-CLV3p16 or LHEEL-CLV3p17 at concentrations of 10 nM, 30 nM, 100 nM, or 1 μM (Figure 4). The results showed that CLV2p12 was active at the concentration of 10 nM, EEL-CLV3p15 was active at 100 nM, while L-CLV3p13 and HEEL-CLV3p16 were not active even at 1 μM, suggesting that these additional N-terminal Leu, Glu-Glu-Leu or His-Glu-Glu-Leu residues caused severe damages to their activities, Strikingly, LHEEL-CLV3p17 was equally active as CLV3p12, i.e. functional at 10 nM. Together with the effective cleavage observed above, we believe that the additional 5 residues in LHEEL-CLV3p17 lead to effective release of CLV3p12, and hence the equivalent activity as CLV3p12.

### Leu69 is critical for the cleavage and activity in synthetic peptide

Previous studies suggest that PSK and RALF are produced through the cleavage after a Leu residue by endopeptidases in *Arabidopsis*[43,44]. To examine if the Leu69 adjacent to the N-terminus of the CLV3 peptide is involved in cleavage, we synthesized an Ala-substituted peptide, LHEEA-CLV3p17, and used MALDI-Tof MS to examine its cleavage after inoculation with seedlings *in vitro*. Interestingly, although removal of a C-terminal residue was observed, no internal cleavage was detected (Figure 3F), suggesting that the Leu69Ala substitution had compromised the internal cleavage in the peptide. We also examined the activity of the LHEEA-CLV3p17 peptide in root assays, and observed that the substitution led to a significantly reduced activity in terminating RMs (Figure 4). These results together indicate that the N-terminal flanking Leu69 is critical for the internal cleavage, and subsequently for the activity of the peptide.

### Leu69 is important for the optimal activity of CLV3 fusion proteins

**Figure 5:**
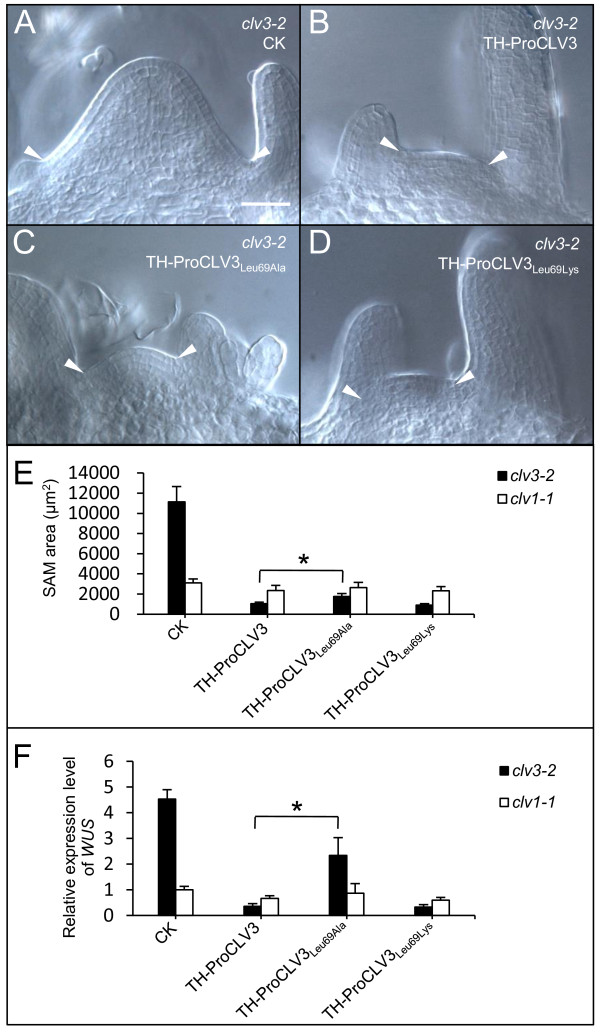
***In vitro *SAM assays using TH-ProCLV3**_**Leu69Ala **_**or TH-ProCLV3**_**Leu69Lys**_**. (A-D)** SAMs of *clv3-2* after treatment with 1 μM TH-ProCLV3 **(B)**, TH-ProCLV3_Leu69Ala_**(C)** or TH-ProCLV3_Leu69Lys_**(D)**, as compared to control samples without fusion proteins **(A)**. The arrowheads indicate margins of the SAMs. The bar in **A** = 50 μm for **A** to **D**. **(E)** Areas of SAMs of 8-d-old *clv3-2* and *clv1-1* seedlings treated with or without fusion proteins (n = 8). Error bar = ± SD. **(F)** qRT-PCR analyses of *WUS* expression in 8-d-old *clv3-2* and *clv1-1* shoot apices treated with or without fusion protein. The average expression level of *WUS* in *clv1-1* without the fusion protein was normalized to 1, and values for other samples represent as relative ratios. Error bar = ± SD of 3 independent biological repeats. The asterisks in **E** and **F** indicate significant differences (*P* < 0.01 by Student’s *t*-test) between TH-ProCLV3- and TH-ProCLV3_Leu69Ala_-treated *clv3-2* seedlings.

To evaluate if the Leu69 is also important for the TH-ProCLV3 fusion protein, we performed an Ala substitution to produce a TH-ProCLV3_Leu69Ala_ fusion protein in *E. coli*. The activity of TH-ProCLV3_Leu69Ala_ in restricting the size of SAMs was examined *in vitro*. The results showed that SAMs in TH-ProCLV3_Leu69Ala_-treated *clv3-2* seedlings were significantly larger than those treated with TH-ProCLV3 (Figure 5B, C, and E). Of course compared to non-treated *clv3-2* seedlings (Figure 5A) they are still smaller. It is evident that the Leu69Ala substitution in TH-ProCLV3_Leu69Ala_ has partially compromised its activity in SAMs.

The commonly known subtilase recognition site is Lys/Arg-Arg [40]. Although in most CLE members of *Arabidopsis* the residue located before the conserved Arg of the 12-AA CLE motif is Lys (Additional file 3), for CLV3 the residue is Leu. We examined if a Leu69Lys substitution hampered the activity of the fusion protein. TH-ProCLV3_Leu69Lys_ was produced in *E. coli* and applied to *clv3-2* seedlings *in vitro*. As expected, no significant difference was observed between the sizes of SAMs treated with TH-ProCLV3_Leu69Lys_ and those treated with TH-ProCLV3 (Figure 5B, D, and E), suggesting that the Leu69Lys substitution did not affect the activity. Additionally, we performed *in vitro* SAM assays in *clv1-1*, and observed no significant differences among samples treated with TH-ProCLV3, TH-ProCLV3_Leu69Ala_, TH-ProCLV3_Leu69Lys,_ and non-treated ones (Figure 5E), implying that the functions of TH-ProCLV3_Leu69Ala_ and TH-ProCLV3_Leu69Lys_ fusion proteins in SAMs are CLV1-dependent, as the CLV3 peptide [34].

To address if these fusion proteins are able to repress the *WUS* expression in SAMs, qRT-PCR analyses were performed with cDNAs from shoot apices after treatments with various fusion proteins. As shown in Figure 5F, the expression level of *WUS* in shoot apices of TH-ProCLV3-treated *clv3-2* seedlings was reduced to about 10% of those non-treated ones. A similar result was obtained in *clv3-2* seedlings treated with TH-ProCLV3_Leu69Lys_ (Figure 5F), confirming that the Leu69Lys substitution did not impair the activity of the fusion protein. In contrast, the expression level of *WUS* in the TH-ProCLV3_Leu69Ala_-treated *clv3-2* seedlings was only about 50% of the untreated ones (Figure 5F). The fact that replacement of Leu69 by Ala, but not by Lys, damaged the activity of CLV3 suggests that, although the Leu69 is not part of the mature CLV3 peptide, it is important for the optimal activity in repressing the *WUS* expression in SAMs.

### Leu69 is critical for the cleavage of CLV3 fusion proteins

**Figure 6:**
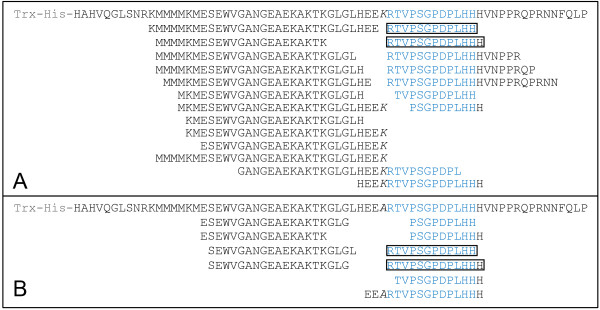
**Peptide fragments released from TH-CLV3**_**Leu69Ala **_**and TH-CLV3**_**Leu69Lys **_**after incubations.** Fragments released from TH-CLV3_Leu69Lys_**(A)** and THCLV3_Leu69Ala_**(B)** are aligned below corresponding fusion protein sequences, as identified with Q-Tof MS/MS analyses of medium samples. Substituted AAs are in italics.

To examine if the Leu69Ala and Leu69Lys substitutions in fusion proteins, namely TH-ProCLV3_Leu69Ala_ and TH-ProCLV3_Leu69Lys_, respectively, led to compromised cleavages, Q-Tof MS/MS analyses were performed on medium samples collected after co-cultivations of these fusion proteins with wildtype seedlings. The TH-ProCLV3_Leu69Lys_ showed a similar peptide profile as seen in the TH-ProCLV3 (Figure 2 and Figure 6A), producing expected CLV3p12 and CLV3p13, and peptides with a C-terminal Lys69. However, for TH-ProCLV3_Leu69Ala_, although both the CLV3p12 and CLV3p13 were detected, no peptide fragment with C-terminal Ala69 was detected (Figure 6B), suggesting that the Leu69Ala substitution may have compromised the cleavage efficiency of the fusion protein or decreased the stability of peptides produced. These results together indicate that both the Leu-Arg and Lys-Arg junctions can be recognized and cleaved by enzymes released by *Arabidopsis* seedlings.

### 5-AA N-terminal flanking sequence is required for proper cleavage of CLE1

To address if the requirement of 5-AA residues in the proper cleavage of CLV3 is generic to other CLE peptides, we synthesized CLE1 peptides with either 0, 1, or 5 N-terminal flanking residues: CLE1p12, M-CLE1p13 and FNESM-CLE1p17, respectively. CLE1 was selected as it complements the *clv3-1* mutant phenotype when expressed under the control of CLV3 regulatory elements in transgenic plants [35]. Within the 5-AA N-terminal flanking sequence, only the Glu (E) residue is conserved between CLV3 and CLE1 (Additional file 3). After 3-d incubation with L*er* seedlings, gradual trimmings of terminal residues were observed in both CLE1p12 and M-CLE1p13, while an internal cleavage was detected between Met (M) and Arg (R) in FNESM-CLE1p17, releasing CLE1p12 (Additional file 4). This suggested that the 5-AA motif is required for proper cleavage of CLE1. As expected, when *in vitro* activity assays were performed in *clv3-2* seedlings, FNESM-CLE1p17 exhibited a similar activity as the CLE1p12 in restricting the sizes of SAMs, while M-CLE1p13 was about 100-fold less active (Additional file 5).

## Discussion

Although it has been shown in *Arabidopsis* that CLV3 acts as a peptide ligand to regulate the number of stem cells in SAMs [21,29,30], how the peptide is generated from the proprotein and if a particular sequence motif is required for the cleavage remain largely unknown. In this study using MS-based analyses, we identified two internal cleavage sites in CLV3 fusion protein produced in *E. coli*, one before Met39 and another one before Arg70. Synthetic peptides with 1, 3 or 4 extra AAs flanking the N-terminus of the CLV3p12 showed greatly reduced activities in terminating RMs *in vitro,* while a peptide with 5 extra AAs restored the activity completely. Peptidomic studies showed that CLV3 and its orthologous CLE1 peptides with 5-AA N-terminal extension exhibited normal cleavage, while shorter extensions hampered the cleavage.

### Two internal cleavage sites identified in CLV3

Peptide hormones are important signal molecules in endocrinal and neural signal transductions in animals [1-4]. They are usually generated from larger protein precursors through post-translational processing by SBT family endopeptidases [39,40]. Among 56 SBT endopeptidases identified in the Arabidopsis genome, 46 of them carry a secretory signal peptide [42]; Two of them, AtSBT1.1 and AtSBT6.1, are involved in PSK4 and RALF23 processing, respectively [43,44]. *CLV3* encodes a 96-AA preprotein and acts as a 12- and/or 13-AA peptide with hydroxylation and glycosylation modifications [29,30]. We speculated that, if CLV3 is processed by secreted enzyme(s), it should be possible to detect such enzymatic activities *in vitro*. We produced the TH-CLV3 fusion protein in *E. coli* and showed it is functional in restoring the *clv3* defect in SAMs *in vitro* through a CLV1-dependent pathway. An *in vitro* cleavage assay was subsequently developed to demonstrate that the TH-ProCLV3 fusion protein was efficiently cleaved 24 hr after inoculation with *Arabidopsis* seedlings, releasing both CLV3p12 and CLV3p13 peptides. After alignments of all fragments released from the CLV3 fusion protein, we confirmed a previously reported cleavage site located before Arg70 [36] and found a novel cleavage one before Met39. As Met39 is located in a region that is not required for the CLV3 function [28], the cleavage might not be functionally relevant.

### The N-terminal junction region is critical for CLV3 cleavage

Previous studies have shown that the Leu69 residue is moderately and Arg70 is highly important for the CLV3 function *in vivo*[33], although Leu69 is not part of the CLV3 mature peptide [29,30]. The involvement of Arg70 in CLV3 processing *in vitro* has been reported [36]. To evaluate the importance of the Leu69 in cleavage, we introduced either Leu69Ala or Leu69Lys substitutions to the TH-ProCLV3 fusion protein and examined their efficiencies in cleavage and subsequently activities in restricting the size of SAMs. We observed that TH-ProCLV3_Leu69Lys_ was cleaved in a similar manner as TH-ProCLV3, while specific cleavages were compromised in TH-ProCLV3_Leu69Ala_, suggesting that enzymes released by *Arabidopsis* seedlings can cleave both the Lys-Arg and Leu-Arg junctions. To be noted, the cleavage in the Leu-Arg junction has not been reported so far in peptide processing in animals. Among CLE family members, although the N-terminal Arg residue in the CLE motif is highly conserved, the residue immediately before the Arg is less conserved (Additional file 3). In Medicago a cleavage at a upstream site before the conserved Lys-Arg junction has been reported [38]. For PSK4 and RALF23, instead of the dibasic Arg-Arg site located upstream, cleavages were detected at the Leu-His and Leu-Ala junctions, respectively [43,44]. It is possible that endopeptidases in plants have more diverse cleavage sites than those in animals.

### The N-terminal flanking sequence of CLV3 is critical for efficient cleavage

Furin is the first SBT discovered in mammals [55]. A 4-AA sequence, Arg-Asn-Lys-Arg, flanking the N-terminus of ectodysplasin-A is essential for its processing by furin [56]. In *Arabidopsis*, six PSK members with different sequences before the cleavage site show different efficiencies of cleavage by AtSBT1.1 [43]. For CLV3 it has been shown previously that addition of a C-terminal His82 to the CLV3 peptide did not have significant effect on its activity in terminating RMs *in vitro*, while addition of an N-terminal Leu69 compromised the activity greatly [31]. In this study we examined effects of adding different numbers of AA residues to the N-terminus of the CLV3 peptide, and observed that peptides with either 1, 3, or 4 additional residues showed reduced activities in terminating RMs. However, the LHEEL-CLV3p17 with a 5-AA extension showed the same activity as the CLV3p12. Further peptidomic analyses revealed that peptides with 1-, 3- or 4-AA extensions were not processed properly, while the one with 5-AA additional residues was. The positive correlation between peptide activity and effective cleavage in LHEEL-CLV3p17 suggests that the 5-AA motif flanking the N-terminus is required for the recognition and proper cleavage to release the functional CLV3 peptide. Consistent with this hypothesis, we showed that the Leu69Ala substitutions in the LHEEA-CLV3p17 peptide and TH-ProCLV3_Leu69Ala_ fusion protein led to reduced activities in terminating RMs and compromised internal cleavage.

With evidence obtained so far, we believe that the length of the N-terminal flanking sequence is more important than the AA identities, with the following reasons: 1) our previous alanine substitution experiments in the flanking residues of CLV3 showed that individual AA in this region contribute very little to CLV3 function *in vivo*[33]; 2) alignment of all CLE proteins encoded in the *Arabidopsis* genome showed very little conservation in the 5-AA flanking region (Additional file 3); 3) CLE1 shares only one AA (Glu) with CLV3 within the 5-AA motif and is able to complement *clv3-1* when expressed under the CLV3 regulatory elements [35]; 4) synthetic CLE1 peptide with the 5-AA N-terminal extension was cleaved properly when inoculated with *Arabidopsis* seedlings. Most likely the 5-AA flanking motif serves as an endopeptidase recognition and/or binding domain for CLV3 and CLE1 cleavages.

## Conclusions

We developed an *in vitro* seedling assay to analyze peptide cleavages and activities in shoot and root meristems in parallel. Using the assay we showed that the maximal activity of the CLV3 requires a proper cleavage between Leu69 and Arg70, and the cleavage requires a recognition domain with at least 5-AA flanking its N-terminus. These findings may help to elucidate the cleavage and function of other peptide hormones, and to identify enzymes involved in peptide processing.

## Methods

### Molecular cloning

The coding region of *CLV3* [GenBank: NM_001124926.1] without the signal peptide was amplified by PCR from cDNA prepared from inflorescences of *Arabidopsis thaliana* (Columbia-0) using primers 5’-GGGGTACCCCCATGCTCA CGTTCAAG-3’ and 5’-GGAATTCCTCAAGGG AGCTGAAAGTTGTTTCT-3’. The PCR product was then cloned into *pEASY-T1* vector (TransGen, Beijing, China), digested with *EcoR*I and *Kpn*I, and subcloned in-frame into *pET-48b*(*+*) (Novagen, Germany) to fuse with the Trx-His tandem tags to generate *TH-ProCLV3*.

To perform AA substitutions, a Fast Mutagenesis System kit (TransGen, Beijing, China) was used to introduce point mutations to *TH-ProCLV3* using primers 5’-TTAGGACTACATGAAGAGGCAAGGACTGTT-3’ and 5’-GCCTCTTCATG TAGTCCTAAACCCTTCGTC-3’, and 5’-TTAGGACTACATGAAGAGAAAA GGACTGTT-3’ and 5’-TTCTCTTCATGTAGTCCTAAACCCTTCGTC-3’ to generate *TH-ProCLV3*_*Leu69Ala*_ and *TH-ProCLV3*_*Leu69Lys*_, respectively, by following the manufacturer’s protocol.

### Fusion protein production and peptide synthesis

Constructs of *TH-ProCLV3, TH-ProCLV3*_*Leu69A*_ and *TH-ProCLV3*_*Leu69Lys*_ were transformed to *E. coli* strain BL21(DE3) individually, and expressions of fusion proteins were induced by 0.1 mM isopropyl β-D-1-thiogalactopyranoside for 16 hr at 16°C. Bacteria were collected by centrifugation and lysed via ultrasonication before the fusion proteins were affinity purified using Ni-NTA agarose (QIAGEN, Germany) according to the manufacturer’s manual. The eluted proteins were dialyzed using a 7-kD retaining dialysis bag and quantified using a Bradford Protein Quantification Kit (Biomed, Beijing, China).

All peptides used in this study were synthesized with over 80% purity (AuGCT, Beijing, China), dissolved in 50 mM sodium phosphate buffer (pH 6.0), and sterilized using a 0.22 μm filter (Millipore, Germany).

### *In vitro* activity assays

Seeds of *clv1-1, clv2-1* and *clv3-2* (all in L*er* background), Col-0 and L*er* were gas-sterilized as reported previously [33], and imbibed in sterilized distilled water at 4°C in dark for 2 days before plating. *In vitro* root and SAM assays were performed as described [17,28].

### qRT–PCR analyses

Total RNA was extracted using a Plant Total RNA Isolation Kit (TIANGEN, Beijing, China) from approximately 30 shoot apices excised from 8-d-old seedlings cultured in fusion protein-containing liquid media, and then reverse transcribed to cDNA with the First Strand cDNA Synthesis Kit ReverTra Ace-α (TOYOBO, Japan). qRT–PCR was performed in a Rotor-Gene 3000 thermocycler (Corbett, Australia) with the SYBR Premix ExTaq II kit (TaKaRa, Dalian, China), and the relative expression levels of the *WUS* [GenBank: NM_127349.3] were normalized against the internal control *EIF4A1* [GenBank: NM_001084679.1] and calculated using the Rotor-Gene 6 software v 6.0 (Corbett, Australia) according to the 2^-∆∆CT^ method [57]. Primer pairs of 5’-GCTCCTCTTAACCCAAAGGC-3’ and 5’-CACACCATCACCAGAATCCAGC -3’, and 5’-TTCTCTGCGACAATGCCTC-3’ and 5’-GCTTCCAGTCTTCTTTC TCCAC-3’ were used to amplify *WUS* and *EIF4A1*, respectively.

### *In vitro* cleavage assay

Gas-sterilized and cold-treated seeds were pre-germinated for 36 hr on half-strength Murashige and Skoog basal salts medium (Sigma-Aldrich, USA), pH 5.8, plus 1% sucrose, 0.5 g/L MES (Merck, Germany) and 1.5% agar. Five seedlings were then transferred to Eppendorf tubes containing 200 μL medium with 10 μM fusion proteins or peptides. These tubes were then placed on a roller bank and cultured at 22°C under a 16 hr light/8 hr dark cycle. Ten μL of media was collected at different time points.

### Mass spectrometry analyses

For Q-Tof MS/MS analyses, medium samples were analyzed with a Triple TOF™ 5600 Q-Tof Micro MS/MS (AB SCIEX, USA) equipped with a CapLC high performance liquid chromatography (Waters, USA) using a fused silica microcapillary column (10 cm) with an internal diameter of 75 μm. Columns were packed with C18 reversed phase resin (GEAgel C18 SP-300-ODS-AP; particle size, 5 μm; pore size, 300 Å; Jinouya, Beijing, China). Separation was achieved using a gradient made from solution A (4% acetonitrile (ACN):96% water, containing 0.1% formic acid) to 50% solution B (80% ACN:20% water, containing 0.1% formic acid) over 70 min and to 100% ACN in 10 min, at a flow rate of 300 nL/min. The flow rate from pumps A and B was 2.5 μL/min, and reduced to approximately 300 nL/min. The MS was operated in a positive ion mode with a source temperature of 80°C and a cone gas flow of 10 L/hr. A voltage of 3 kV was applied to the nanoflow probe tip. MS and MS/MS spectra were acquired in an automated, data-dependent mode, and all data were processed using MassLynx version 4.0 software to generate “.DTA” files. The instrument was calibrated with a multi-point calibration using selected fragment ions that resulted from the CID of Glu-fibrinopeptide B (+2 ion, m/z 785.8, Sigma-Aldrich, USA). Sequences of peptides were obtained from Turbo SEQUEST searching with Bioworks version 3.2 software (Thermo, USA) using individual peptide databases made for TH-ProCLV3, TH-ProCLV3_Leu69Ala_ and TH-ProCLV_Leu69Lys_, followed by the post-filter Xcorr value > 1.5 and DeltaCn value > 0.01.

For MALDI-Tof MS analyses, 2 μL medium samples were spotted directly on a stainless steel plate, rinsed with matrix solutions, and air-dried before analyses with an AXIMA-CFR™plus (SHIMADZU, Japan), operated with a pulsed nitrogen laser at 337 nm. One hundred to 150 shots per spectrum were performed on each spot. For protein cleavage assays, sinapinic acid saturated in ACN/0.1% trifluoric acid (TFA) 3:2 (v/v) was used as the matrix solution, and positive-ion mass spectra were acquired in linear, delayed extraction mode. For peptides, α-cyano-4-hydroxycinnamic acid saturated in ACN/0.1% TFA 1:1 was used as the matrix, and data were acquired in the reflectron mode. The analyzer was externally calibrated with aldolase (Sigma-Aldrich, USA) for protein cleavage assays and with a mixture of Bradykinin fragment 1-7 (Sigma-Aldrich, USA), P_14_R (Sigma-Aldrich, USA) and ACTH fragment 18-39 (Sigma-Aldrich, USA) for peptides.

## Competing interests

The authors declare that they have no competing interests.

## Authors’ contributions

TTX designed and performed the experiments and wrote the manuscript; SCR assisted with some experiments; CML and XFS guided the study and revised the manuscript. All authors read and approved the manuscript.

## Supplementary Material

Additional file 1 **Cleavages of the TH-ProCLV3 fusion protein after co-cultivation with L** ***er *** **seedlings for 0, 12, 24 and 48 hrs, as showed by MALDI-Tof MS analyses.** Arrows indicate the peak of TH-ProCLV3. Note the most abundant small peptides (marked by red brackets) were detected after the 24-hr inoculation. Click here for file

Additional file 2 **Cleavages of the LHEEL-CLV3p17 peptide after co-cultivation with L** ***er *** **seedlings for 0, 1, 2 and 3 d, as showed by MALDI-Tof MS analyses.** Peptide sequences, defined based on their accurate masses, are showed near corresponding peaks. The core CLE motif of CLV3 is shown in blue. Click here for file

Additional file 3 **Alignment of core CLE motifs and five N-terminal flanking residues for all CLE proteins in *Arabidopsis.*** The core CLE motif (framed) and five N-terminal flanking residues of CLE proteins from Arabidopsis are aligned. The Lys residue flanking the N-terminus of the CLE motif is highlighted in red. Click here for file

Additional file 4 ***In vitro *** **cleavage assay of CLE1 peptides with different N-terminal extensions.** Mass spectra of CLE1p12 (A), M-CLE1p13 (B) and FNESM-CLE1p17 (C) after 3-d incubations with L*er* seedlings. Sequences of the original peptides are shown at the upper right corner of each figure, where detected cleavage sites are marked by black arrowheads. Sequences of individual fragments are labeled near corresponding peaks. Original peptide peaks are marked by blue arrowheads, while CLE1p12 by red arrowheads. Click here for file

Additional file 5 ***In vitro *** **activity assay of CLE1 peptides with different N-terminal extensions.** (A-D) SAMs of *clv3-2* after treatments with CLE1p12 (B), M-CLE1p13 (C) or FNESM-CLE1p17 (D), as compared to the control without peptide (A). The arrowheads indicate margins of the SAMs. The bar in A = 50 μm for A to D. (E) Average SAM areas of *clv3-2* seedlings (n = 16 for all treatments) after 8-d incubations in media containing CLE1 peptides with different N-terminal extensions (showed above). Error bar = ± SD. Average SAM areas with significant difference from the non-treated one (*P* < 0.01 by Student’s *t*-test) are marked with asterisks. Click here for file
