# Supplementary material for: The sequence flanking the N-terminus of the CLV3 peptide is critical for its cleavage and activity in stem cell regulation in Arabidopsis
Source: BMC Plant Biol. 2013 Dec 27;13:225. doi: 10.1186/1471-2229-13-225 (PMC3878228; doi:10.1186/1471-2229-13-225)
Supplement: Additional file 1 — Cleavages of the TH-ProCLV3 fusion protein after co-cultivation with L er seedlings for 0, 12, 24 and 48 hrs, as showed by MALDI-Tof MS analyses. Arrows indicate the peak of TH-ProCLV3. Note the most abundant small peptides (marked by red brackets) were detected after the 24-hr inoculation. [file 1471-2229-13-225-S1.pdf]

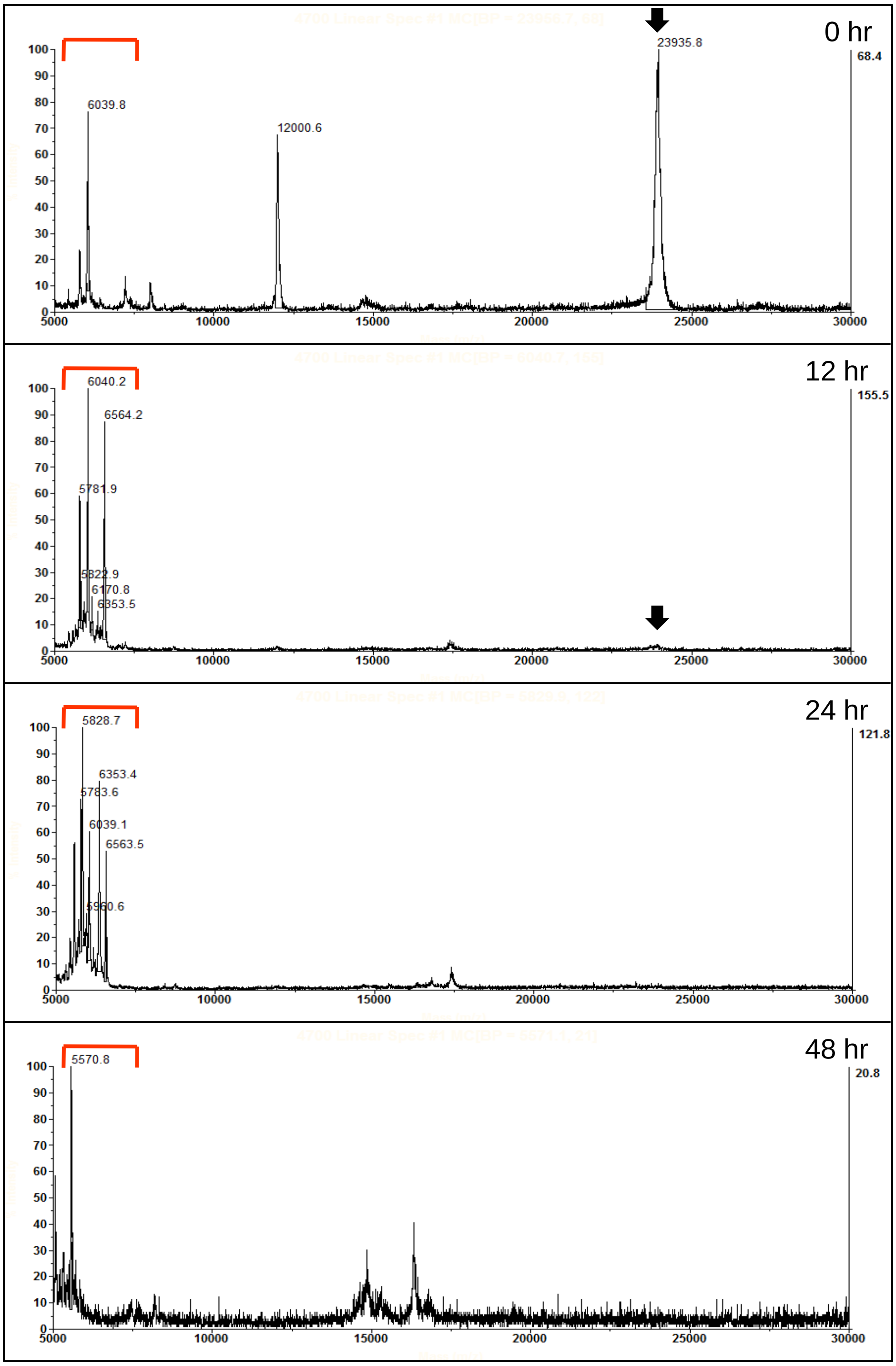

Additional file 1. Cleavages of the TH-ProCLV3 fusion protein after co-cultivation with L*er* seedlings for 0, 12, 24 and 48 hrs, as showed by MALDI-Tof MS analyses

Arrows indicate the peak of TH-ProCLV3. Note the most abundant small peptides (marked by red brackets) were detected after the 24-hr inoculation.
