# Supplementary material for: The sequence flanking the N-terminus of the CLV3 peptide is critical for its cleavage and activity in stem cell regulation in Arabidopsis
Source: BMC Plant Biol. 2013 Dec 27;13:225. doi: 10.1186/1471-2229-13-225 (PMC3878228; doi:10.1186/1471-2229-13-225)
Supplement: Additional file 2 — Cleavages of the LHEEL-CLV3p17 peptide after co-cultivation with L er seedlings for 0, 1, 2 and 3 d, as showed by MALDI-Tof MS analyses. Peptide sequences, defined based on their accurate masses, are showed near corresponding peaks. The core CLE motif of CLV3 is shown in blue. [file 1471-2229-13-225-S2.pdf]

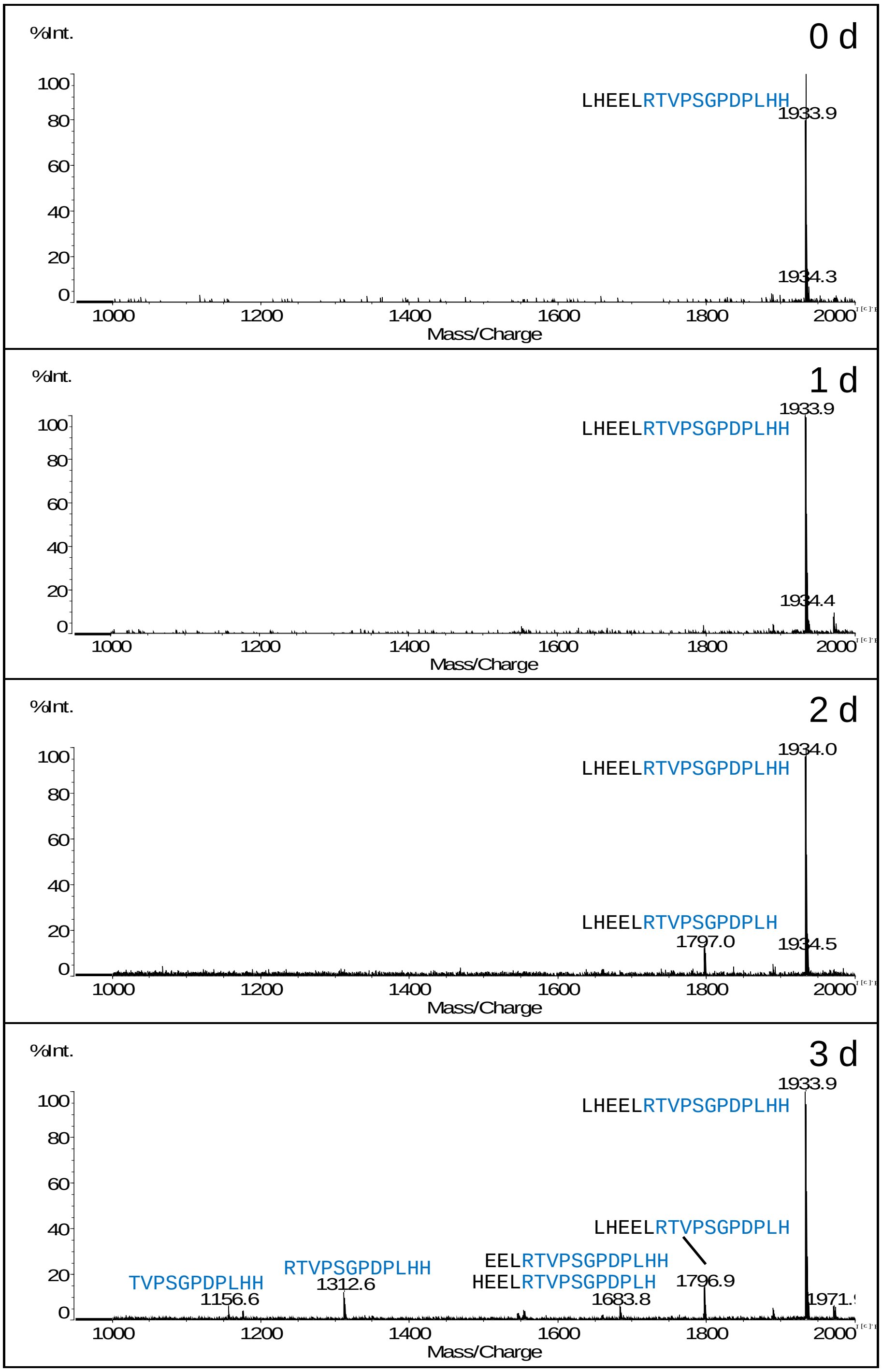

Additional file 2. Cleavages of the LHEEL-CLV3p17 peptide after co-cultivation with L*er* seedlings for 0, 1, 2 and 3 d, as showed by MALDI-Tof MS analyses

Peptide sequences, defined based on their accurate masses, are showed near corresponding peaks. The core CLE motif of CLV3 is shown in blue.
