# Supplementary material for: The sequence flanking the N-terminus of the CLV3 peptide is critical for its cleavage and activity in stem cell regulation in Arabidopsis
Source: BMC Plant Biol. 2013 Dec 27;13:225. doi: 10.1186/1471-2229-13-225 (PMC3878228; doi:10.1186/1471-2229-13-225)
Supplement: Additional file 3 — Alignment of core CLE motifs and five N-terminal flanking residues for all CLE proteins in Arabidopsis. The core CLE motif (framed) and five N-terminal flanking residues of CLE proteins from Arabidopsis are aligned. The Lys residue flanking the N-terminus of the CLE motif is highlighted in red. [file 1471-2229-13-225-S3.pdf]

```
CLV3      --LHEELRTVPSGPDPLHH--
CLE1      --FNESMRLSPGGPDPRHH
CLE2      --GKSPERLSPGGPDPQHH
CLE3      --TLDSKRLSPGGPDPRHH
CLE4      --TLDSKRLSPGGPDPRHH
CLE5      --LVSSDRVSPGGPDPQHH
CLE6      --LVDSERVSPGGPDPQHH
CLE7      --QNEVDRFSPGGPDPQHH--
CLE8      --LFRTMRRVPTGPNPLHH--
CLE9      --YGVDKRLVPSGPNPLHN
CLE10     --YGVEKRLVPSGPNPLHN
CLE11     --YNDEERVVPSGPNPLHH
CLE12     --YGVEKRRVPSGPNPLHH
CLE13     --YGVEKRLVPSGPNPLHH
CLE14     --VGASARLVPKGPNPLHNK
CLE16     --YKDDKRLVHTGPNPLHN
CLE17     --YGDDKRVVHTGPNPLHN
CLE18     --LIGVDRQIPTGPDPLHN--
CLE19     --ALDSKRVIPTGPNPLHNR
CLE20     --ILPDKRKVKTGSNPLHNKR
CLE21     --EEEEKRSIPTGPNPLHNK
CLE22     --FEDGKRRVFTGPNPLHNR
CLE25     --FHVSKRKVPNGPDPIHN--
CLE26     --YVASKRKVPRGPDPIHN--
CLE27     --ISESKRIVPSCPDPLHN
CLE40     --NEVEERQVPTGSDPLHH--
CLE41     --FGNDAHEVPSGPNPISN
CLE42     --IGANEHGVPSGPNPISNR
CLE43     --FEDSNRRIVSSPDRLHN
CLE44     --FRAEAHEVPSGPNPISN
CLE45     --FKSSKRRVRRGSDPIHN--
CLE46     --EEKKWHKHPSGPNPTGN--
```

Additional file 3. Alignment of core CLE motifs and 5 N-terminal flanking residues for all CLE proteins in *Arabidopsis*

The core CLE motif (framed) and 5 N-terminal flanking residues of CLE proteins from Arabidopsis are aligned. The Lys residue flanking the N-terminus of the CLE motif is highlighted in red.
