# Supplementary material for: The sequence flanking the N-terminus of the CLV3 peptide is critical for its cleavage and activity in stem cell regulation in Arabidopsis
Source: BMC Plant Biol. 2013 Dec 27;13:225. doi: 10.1186/1471-2229-13-225 (PMC3878228; doi:10.1186/1471-2229-13-225)
Supplement: Additional file 4 — In vitro cleavage assay of CLE1 peptides with different N-terminal extensions. Mass spectra of CLE1p12 (A), M-CLE1p13 (B) and FNESM-CLE1p17 (C) after 3-d incubations with Ler seedlings. Sequences of the original peptides are shown at the upper right corner of each figure, where detected cleavage sites are marked by black arrowheads. Sequences of individual fragments are labeled near corresponding peaks. Original peptide peaks are marked by blue arrowheads, while CLE1p12 by red arrowheads. [file 1471-2229-13-225-S4.pdf]

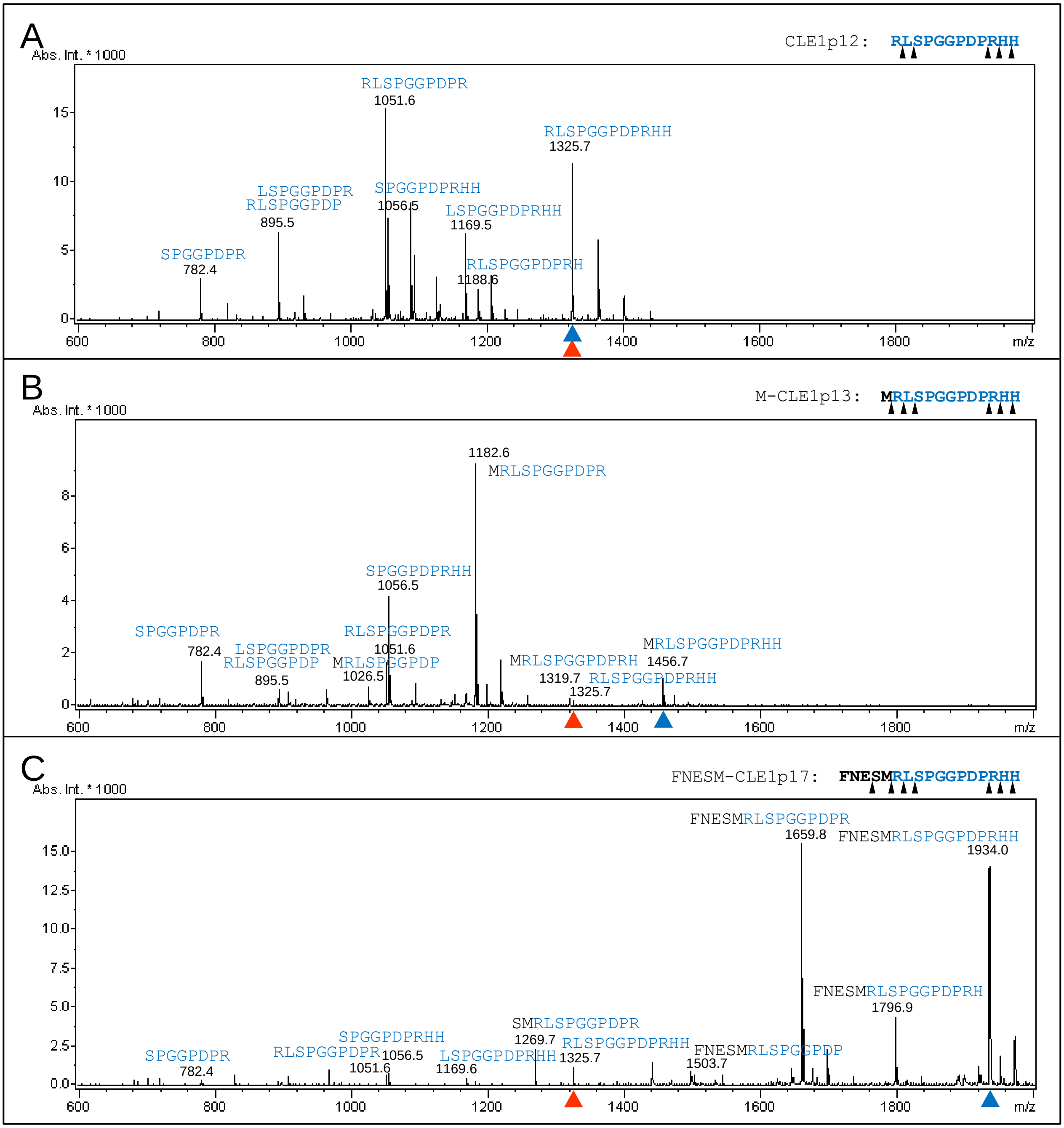

Additional file 4. ***In vitro*** **cleavage assay of CLE1 peptides with different N-terminal extensions**

Mass spectra of CLE1p12 **(A)**, M-CLE1p13 **(B)** and FNESM-CLE1p17 **(C)** after 3-d incubations with L*er* seedlings. Sequences of the original peptides are shown at the upper right corner of each figure, where detected cleavage sites are marked by black arrowheads. Sequences of individual fragments are labeled near corresponding peaks. Original peptide peaks are marked by blue arrowheads, while CLE1p12 by red arrowheads.
