# Supplementary material for: The sequence flanking the N-terminus of the CLV3 peptide is critical for its cleavage and activity in stem cell regulation in Arabidopsis
Source: BMC Plant Biol. 2013 Dec 27;13:225. doi: 10.1186/1471-2229-13-225 (PMC3878228; doi:10.1186/1471-2229-13-225)
Supplement: Additional file 5 — In vitro activity assay of CLE1 peptides with different N-terminal extensions. (A-D) SAMs of clv3-2 after treatments with CLE1p12 (B), M-CLE1p13 (C) or FNESM-CLE1p17 (D), as compared to the control without peptide (A). The arrowheads indicate margins of the SAMs. The bar in A = 50 μm for A to D. (E) Average SAM areas of clv3-2 seedlings (n = 16 for all treatments) after 8-d incubations in media containing CLE1 peptides with different N-terminal extensions (showed above). Error bar = ± SD. Average SAM areas with significant difference from the non-treated one (P < 0.01 by Student’s t-test) are marked with asterisks. [file 1471-2229-13-225-S5.pdf]

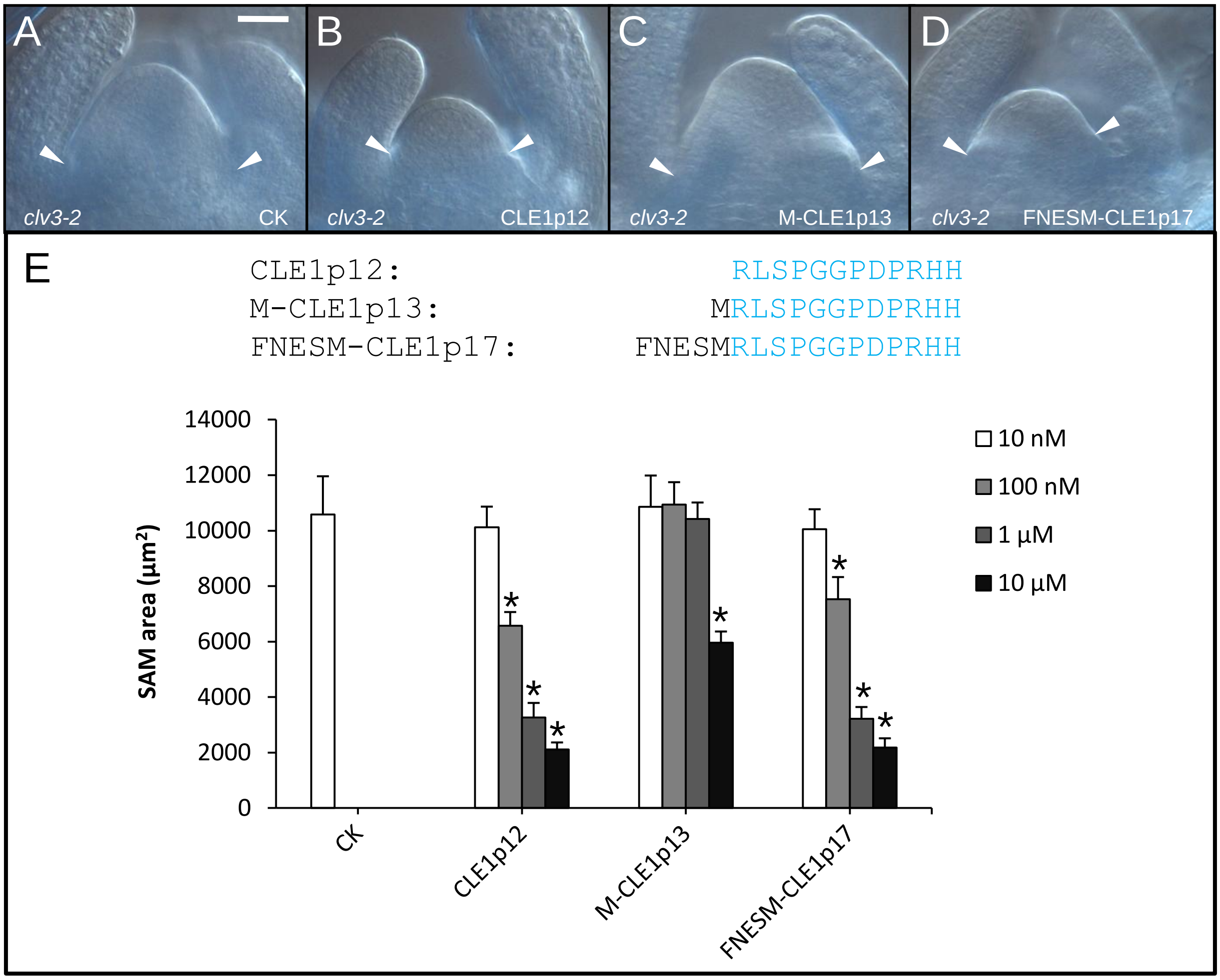


**Additional file 5. *In vitro* activity assay of CLE1 peptides with different N-terminal extensions**

**(A-D)** SAMs of *clv3-2* after treatments with CLE1p12 **(B)**, M-CLE1p13 **(C)** or FNESM-CLE1p17 **(D)**, as compared to the control without peptide **(A)**. The arrowheads indicate margins of the SAMs. The bar in **A** = 50 µm for **A** to **D**. **(E)** Average SAM areas of *clv3-2* seedlings (n = 16 for all treatments) after 8-d incubations in media containing CLE1 peptides with different N-terminal extensions (showed above). Error bar = ± SD. Average SAM areas with significant difference from the non-treated one ($P < 0.01$ by Student's *t*-test) are marked with asterisks.
